# Characteristics and treatment preferences of individuals with opioid use disorder seeking to transition from buprenorphine to extended‐release naltrexone in a residential setting

**DOI:** 10.1111/ajad.13264

**Published:** 2022-02-09

**Authors:** Paolo Mannelli, Antoine B. Douaihy, Sarah C. Akerman, Anna Legedza, James Fratantonio, Abigail Zavod, Maria A. Sullivan

**Affiliations:** ^1^ Department of Psychiatry and Behavioral Sciences Duke University Medical Center Durham North Carolina USA; ^2^ Department of Psychiatry University of Pittsburgh School of Medicine Pittsburgh Pennsylvania USA; ^3^ Alkermes, Inc. Waltham Massachusetts USA; ^4^ Department of Psychiatry Columbia University New York New York USA

## Abstract

**Background and Objectives:**

Treatment for individuals receiving medication for opioid use disorder (MOUD) should follow an informed patient‐centered approach. To better support patient autonomy in the decision‐making process, clinicians should be aware of patient preferences and be prepared to educate and assist patients in transitioning from one MOUD to another, when clinically indicated. This posthoc analysis describes the characteristics of clinical trial participants (NCT02696434) with a history of opioid use disorder (OUD) seeking to transition from buprenorphine (BUP) to extended‐release naltrexone (XR‐NTX).

**Methods:**

The posthoc analysis included adults with OUD currently receiving BUP (≤8 mg/day) and seeking transition to XR‐NTX *(N* = 101) in a residential setting. Baseline participant characteristics and OUD treatment history were reviewed. All patients completed a screening questionnaire that asked about their reasons for seeking transition to XR‐NTX and for choosing BUP.

**Results:**

The most common reasons for initiating a transition to XR‐NTX were “Seeking to be opioid‐free” (63.4%) and “Tired of daily pill taking” (25.7%). Positive predictors of transition included a more extensive BUP treatment history and a history of prescription opioid abuse. Most participants stated they were not aware of XR‐NTX as a treatment option when initiating BUP (78.2%).

**Discussions and Conclusions:**

Patients' reasons for seeking XR‐NTX transition, more extensive BUP treatment history, and a history of prescription opioid abuse, may positively predict outcomes.

**Scientific Significance:**

These findings may assist clinicians in optimizing outcomes of the BUP to XR‐NTX transition and supporting patients to make better informed MOUD decisions.

## INTRODUCTION

For individuals receiving medication for opioid use disorder (MOUD), treatment needs and medication preference may change over the course of their recovery, and it is recommended that treatment decisions follow an informed patient‐centered approach. However, involving patients in the clinical decision‐making process may only occur at a minority of substance use disorder clinics.^1^ To support patient autonomy, it is important that clinicians be aware of expressed patient preferences and be prepared to educate and assist patients, when clinically indicated, in transitioning from one MOUD to another. All US Food and Drug Administration (FDA)‐approved MOUDs (methadone, buprenorphine [BUP], and extended‐release naltrexone [XR‐NTX]) are effective and should be considered by all patients as potential treatment options (https://www.asam.org/Quality-Science/quality/2020-national-practice-guideline).

For individuals on MOUD, a survey study found that 62% (90/145) had high interest in discontinuing treatment; this intention was associated with having discussed this option with a number of different people (e.g., prescribing doctor, family/friend, case manager, general practitioner).^2^ However, barriers to discontinuation include worry about withdrawal symptoms and fear of relapse.^2^, ^3^ For individuals seeking to discontinue BUP, the Substance Abuse and Mental Health Services Administration (https://www.samhsa.gov/, Tip 63) recommends that patients be counseled about the risk of relapse, be monitored during and after BUP dose taper, and be urged to consider antagonist therapy with XR‐NTX. In particular, patient education about the benefits and challenges of discontinuing BUP should include a discussion of the risks of overdose and death and the high rates of relapse observed when patients discontinue BUP without additional MOUD support.^4^, ^5^

XR‐NTX, a once‐monthly injectable medication, is indicated for the prevention of relapse to opioid dependence following opioid detoxification. A clinical trial evaluated 7‐day transition regimens for individuals with a history of opioid use disorder (OUD) currently receiving BUP treatment and seeking to transition from BUP to XR‐NTX; overall, 72% of participants in this study successfully received XR‐NTX.^5^ In this posthoc analysis, we describe the characteristics of participants in this study, including their reasons for seeking to transition from BUP to XR‐NTX.

## METHODS

### Study design

This Phase 3, multicenter, double‐blind, placebo‐controlled, randomized, hybrid residential‐outpatient trial of adults with OUD currently receiving BUP treatment and transitioning from BUP to XR‐NTX (VIVITROL®, Alkermes, Inc.; ClinicalTrials.gov identifier: NCT02696434) was conducted from May 2016 to November 2017.^5^ The study protocol has been described in detail previously.^5^ The study included a 7‐day residential induction onto XR‐NTX (Days 1–7, consisting of standing doses of ancillary medications [clonidine, clonazepam, trazodone], a descending taper of BUP, and transition regimens that included ascending doses of oral NTX vs. placebo), followed by XR‐NTX injection (Day 8), and discharge. Screening (including medical/psychiatric assessment and review of BUP treatment course) was from Days −26 to −6 (outpatient), followed by lead‐in BUP stabilization on ≤4 mg daily. Baseline was on Day 1 (residential).

### Participants

The study participants (*N* = 101) have been characterized previously.^5^ The sample was recruited through radio, print, and digital advertisements, as well as through word‐of‐mouth referrals at sites that offered clinical treatment for OUD. The key inclusion criteria included age 18–60 years, voluntarily seeking treatment for OUD with an interest in transitioning to XR‐NTX, diagnosis of OUD (by *Diagnostic and Statistical Manual of Mental Disorders* [Fifth Edition]) for the prior ≥6 months, history of prescribed BUP (or BUP/naloxone) for the prior ≥3 months, and stable treatment on BUP ≤8 mg/day for ≥30 days. The key exclusion criteria included a history of more than three unsuccessful opioid detoxifications or a history of an accidental drug overdose, a positive urine drug screen for methadone, opiates (other than BUP) or oxycodone at screening (initiation of the BUP lead‐in period), the use of NTX (oral or XR‐NTX) within 90 days before screening, the use of methadone within 30 days before screening, or a history of seizures or anticonvulsant therapy during the last 5 years.^5^

### Participant characteristics

In this posthoc analysis, we reviewed the baseline participant characteristics (previously reported by Comer et al.^5^) and OUD treatment history.

### Participant questionnaire

The relevant portion of the self‐reported screening questionnaire asked participants about (i) their reasons for seeking transition from BUP to XR‐NTX and (ii) their reasons for choosing BUP. All participants completed the questionnaire. Questions and response options are included in the Figure 3 legend.

## RESULTS

### Baseline participant characteristics

As reported by Comer et al.,^5^ the median age of participants was 34.0 years (range 20–57 years), most were male (71/101; 70.3%), most were white (93/101; 92.1%), participants were on BUP dose of 8 mg/day (61/101; 60.4%) or <8 mg/day (40/101; 39.6%), and participants generally had mild baseline withdrawal symptoms (median Clinical Opiate Withdrawal Scale score 3.0, range 0–15; median Subjective Opiate Withdrawal Scale score 2.0, range 0–47), consistent with a condition of stable BUP treatment.

### Treatment history of participants

**FIGURE 1:**
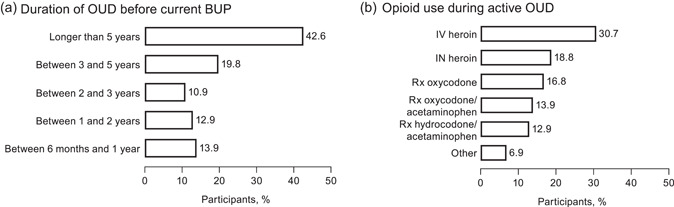
Opioid use of participants seeking to transition from buprenorphine (BUP) to extended‐release naltrexone. (a) Duration of opioid use disorder (OUD) before current BUP course of treatment (at baseline). (b) History of opioid use during active OUD (before study entry). IN, intranasal; IV, intravenous; Rx, prescription

**FIGURE 2:**
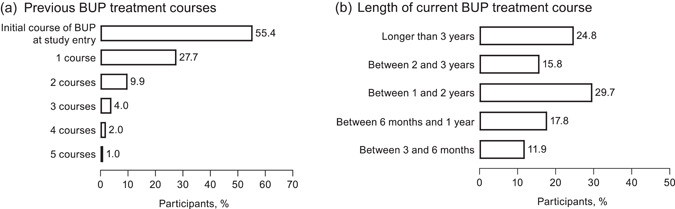
Buprenorphine (BUP) treatment of participants seeking to transition from BUP to extended‐release naltrexone. (a) Number of previous BUP treatment courses (at baseline). (b) Length of current BUP treatment course (at baseline)

Nearly one‐half (43/101; 42.6%) of participants had a history of OUD for more than 5 years (Figure 1a).^5^ The most commonly used opioids before BUP treatment were intravenous heroin, intranasal heroin, and oxycodone (Figure 1b). A majority of participants (56/101; 55.4%) were in their first course of BUP treatment (Figure 2a). Most participants (71/101; 70.3%) had been taking their current course of BUP for at least 1 year (Figure 2b).^5^

At screening, more than one‐third of participants (38/101; 37.6%) reported alcohol and/or illicit drug use during the past week (alcohol, 27/101, 26.7%; cannabis, 33/101, 32.7%; benzodiazepines, 4/101, 4.0%; stimulants, 1/101, 1.0%). In addition, many patients had positive toxicology results from a urine drug test at screening (cannabis, *n* = 35; benzodiazepines, *n* = 28; cocaine, *n* = 9; amphetamine, *n* = 4; methamphetamine, *n* = 1; barbiturates, *n* = 1). Note that participants could have reported use of, or had positive toxicology for, more than one drug.

### Reasons for seeking to transition from BUP to XR‐NTX

**FIGURE 3:**
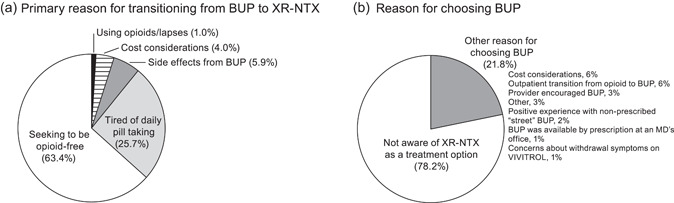
Questionnaire results of participants seeking to transition from buprenorphine (BUP) to extended‐release naltrexone (XR‐NTX) (as per a questionnaire with pre‐populated answer choices). (a) Primary reason for the transition from BUP to XR‐NTX (at screening). Participants were asked, “What is your main reason for wanting to transition from BUP to VIVITROL?” Answer choices consisted of the following: “Seeking to be opioid‐free,” “Tired of daily pill taking,” “Side effects from BUP,” “Still experiencing cravings for opioids,” “Using opioids/lapses while on BUP,” “Work‐related concerns,” “Childcare‐related concerns,” “Transportation is inconvenient,” “Hassle of filling prescriptions,” “Concerns about BUP being lost/stolen,” “BUP requires too many appointments,” “Cost considerations,” or “Other.” The reported “Side effects from BUP” were sweats/chills (*n* = 2), dizziness/lightheadedness (*n* = 1), drowsiness/sleepiness (*n* = 1), other (*n* = 1), and mental slowing (*n* = 1). (b) Awareness of XR‐NTX as a treatment option when BUP was initiated (at screening). Participants were asked “Why did you choose BUP?” Answer choices consisted of the following: “Not aware of VIVITROL at the time,” “Seeking outpatient detox/transition to medication‐assisted treatment,” “Cost considerations,” “Provider encourages BUP over other treatments,” “Positive experience with non‐prescription ‘street’ BUP,” “BUP was available by prescription from doctor's office,” “Concerns about withdrawal symptoms on VIVITROL,” or “Other.” For (a) and (b), only one answer could be selected, and “None of the above” was not included as an option

The most commonly reported reasons for wanting to transition from BUP to XR‐NTX were “Seeking to be opioid‐free” (63.4%) and “Tired of daily pill taking” (25.7%) (Figure 3a). Participants’ reasons for wanting to transition from BUP to XR‐NTX did not differ based on daily BUP dose (8 vs. <8 mg), BUP treatment duration (6 months to 2 years vs. >2 years), history of opioid use (heroin vs. prescription opioids), gender (female vs. male), or age (median age ≤34 vs. >34 years).

### Baseline predictors of successful transition from BUP to XR‐NTX

The likelihood of transition from BUP to XR‐NTX was predicted using descriptive statistics. Of the 101 study participants, 72.3% (*n* = 73) successfully transitioned from BUP to NTX. The responses “Seeking to be opioid‐free” and “Tired of daily pill taking,” represented by 65.8% (48/73) and 25.7% (18/73) of transitioned participants, respectively, were associated with a higher likelihood of transition from BUP to XR‐NTX than any of the reference combination responses (“Cost considerations,” “Side effects from BUP,” “Using opioids/lapses while on BUP”), represented by 9.6% (7/73). Successful transition from BUP to XR‐NTX was seen in 75% of 64 (*n* = 48) study participants who responded, “Seeking to be opioid‐free,” 69.2% of 26 (*n* = 18) study participants who responded, “Tired of daily pill taking,” and 63.6% of 11 (*n* = 7) study participants who responded, “Cost considerations,” “Side effects from BUP,” or “Using opioids/lapses while on BUP.”

In addition, when using a Cox proportional hazards model for time to event (transition from BUP to XR‐NTX is defined as the event) analysis, participants maintained on BUP treatment for 1–2 years (hazard ratio [HR], 1.22) or 2–3 years (HR, 1.19) had a higher likelihood of transition to XR‐NTX than those maintained on BUP for less than a year (reference combination of 3–6 months and 6–12 months). Compared with the reference group with no prior courses of BUP treatment, having had three or more prior courses (HR, 1.33), two prior courses (HR, 1.18), or one prior course (HR, 1.28) of BUP treatment also predicted a higher likelihood of successful transition. By contrast, a history of intravenous heroin use (HR, 0.81) or intranasal heroin use (HR, 0.98) as the primary opioid used before BUP treatment was associated with a lower likelihood of XR‐NTX transition than a history of using prescription opioids. This finding is consistent with other XR‐NTX studies, which have noted that lower severity of opioid dependence is a positive predictor of completing XR‐NTX initiation.^6^

### Awareness of XR‐NTX as a treatment option

More than three‐quarters of participants (79/101; 78.2%) stated that they had not been aware of XR‐NTX as a treatment option when they had first initiated BUP treatment (Figure 3b). Other reasons for choosing BUP as the initial treatment included a decision to avoid inpatient detoxification (“Seeking outpatient detox/transition to medication‐assisted treatment,” 6/101; 5.9%) and “Cost considerations” (6/101; 5.9%).

## DISCUSSION

In this study of individuals receiving BUP and seeking XR‐NTX treatment, participants most commonly had a history of heroin use and a diagnosis of OUD for more than 5 years, were on their first course of BUP, and were seeking to be opioid‐free after at least 1 year of BUP treatment. In addition, more than one‐third of otherwise treatment‐stable individuals (maintained on ≤8 mg/day BUP for at least 30 days, and in BUP treatment 3 months or longer) were using illicit drugs or alcohol at screening, although providing a urine drug test free of opioids (except BUP) was required for study entry.

Patients most likely to achieve a successful transition from BUP to XR‐NTX reported being motivated by seeking to be opioid‐free or to avoid daily pill taking, had currently been treated with BUP for more than 1 year, had received at least one prior course of BUP treatment, and had a history of prescription opioid use before BUP initiation. Identifying the characteristics of patients seeking to transition from BUP to XR‐NTX may inform treatment decisions and help develop effective patient‐centered care practices for OUD. In particular, the finding that most participants lacked awareness of XR‐NTX as a treatment option when initiating BUP highlights the current need for patient education on MOUD options and the importance of fully informed consent when making the decision to initiate treatment. In a study of patient preferences and beliefs about MOUD among patients undergoing opioid detoxification, when all three FDA‐approved medications were described, the most frequently endorsed choice (32%) was XR‐NTX, despite many patients’ lack of prior experience with this medication.^7^ And, consistent with our current findings, among patients who had previously taken BUP, a relatively high proportion (34%) preferred XR‐NTX.^7^

The results of the present study demonstrate that, in the course of long‐term management of OUD, many patients may choose to transition from one MOUD to another. Assessment of patients’ beliefs and goals is important to fostering continued treatment engagement. We found that endorsing a desire to become opioid‐free or to discontinue pill taking, among patients who had been treated with BUP for more than 12 months for prescription opioid misuse, predicted a successful transition from BUP to XR‐NTX. The finding that a past history of prescription opioid use, pre‐dating buprenorphine treatment, predicts successful transition to XR‐NTX extends earlier research findings that a current history of lower severity opioid dependence is a positive predictor of induction from untreated OUD onto XR‐NTX.^6^

Clinicians can play a significant role in facilitating patient‐centered care practices for OUD, particularly when supporting the patient's choice of treatment.^8^ A survey of patients enrolled in BUP treatment found that the decision to seek discontinuation was associated with having engaged in a recent discussion with a clinician.^9^ However, clinicians may not exert equal influence with respect to all MOUD decisions. For example, a semi‐structured interview study of individuals with a history of MOUD found that patients became interested in BUP treatment through peer education; in contrast, patients became interested in XR‐NTX treatment through clinicians.^10^ A patient's personal beliefs about the efficacy and safety of a treatment, or its consistency with the goal of being opioid‐free, may play a major role in MOUD decision‐making.^7^ Personal barriers specific to treatment should also be considered; in addition to a lack of awareness of the medication, the barriers to XR‐NTX treatment often include limited access to medically supervised detoxification and fear of, or lack of interest in, antagonist treatment.^11^

This study is inherently limited as a posthoc analysis. In addition, we collected data at screening and baseline (study entry) from a specific population of patients who were interested in starting antagonist treatment for OUD, as this was a criterion for study entry; only data from participants included in the study are presented. Thus, the findings of this analysis may not be generalizable to all patients with OUD, but are most relevant to the subset of patients with characteristics similar to those in this study (i.e., abstinent from illicit opioids, maintained on or able to taper down to ≤8 mg BUP per day, residential transition). The short duration of this trial (5 weeks) did not allow for long‐term follow‐up to evaluate continued XR‐NTX adherence and opioid abstinence. Finally, self‐report measures of discontinuation reason and medication awareness did not use validated questionnaires and did not offer open‐ended questions or a wider range of possible options, and lack of awareness of XR‐NTX would have limited the ability of participants to address “Concerns about withdrawal symptoms on VIVITROL,” which was one of the response options. However, despite these limitations, and that patient awareness of XR‐NTX may have changed, as well as a recent increase in the use of potent synthetic opioids, the participants' reasons for seeking XR‐NTX treatment were similar to those identified in other clinical populations.^9^

In conclusion, our study highlights the characteristics of clinical trial patients voluntarily seeking transition from BUP to XR‐NTX, as well as those who were successful in this transition. Most patients seeking out this change in course of treatment had not only an extensive (>5 years) history of OUD but also a relatively long (>12 months) period of BUP stabilization, and other substance or alcohol use was common in this clinical population. While the decision to seek transition from BUP to XR‐NTX is consistent with patient autonomy, our findings point to the importance of reviewing the patient's motivation to change treatment (e.g., desire to be opioid‐free, avoidance of daily pill taking) and considering certain features of their drug use history that may suggest a more challenging transition (e.g., a history of heroin use or more severe opioid dependence pre‐dating BUP treatment) and requirement for extra care. If a patient seeks guidance about the timing of the induction, the treatment provider may wish to consider that patients with longer periods of stabilization on BUP (maintained on BUP for 12 months or longer, or currently on their second BUP course), may be more likely to complete the transition. These findings may assist clinicians in their efforts to educate patients and optimize outcomes of the BUP to XR‐NTX transition, to support patients in making better informed MOUD decisions. Importantly, and in alignment with findings from other recent studies,^11^ we propose that a lack of awareness of antagonist therapy by patients (with a relative paucity of XR‐NTX providers and a potential lack of familiarity with antagonist therapy by clinicians) remains a major barrier to initiating treatment with XR‐NTX. This challenge should be addressed by including all treatment options for OUD in clinician and patient education and highlighting the benefits for patients in exercising individual autonomy while remaining engaged with their MOUD treatment.

## CONFLICT OF INTERESTS

Paolo Mannelli, MD, has received consultation fees andgrants from Alkermes and other pharmaceutical companies. Antoine B Douaihy, MD,has participated in advisory boards and received grants from Alkermes. Sarah C.Akerman, MD is an employee and may be a shareholder of Alkermes, Inc. AbigailZavod, MD, MPH; Anna Legedza, ScD; James Fratantonio, PharmD; and Maria A.Sullivan, MD, PhD, are former employees of Alkermes, Inc.

## AUTHOR CONTRIBUTIONS

All authors participated in the interpretation of study results and in the drafting, critical revision, and approval of the final version of the manuscript. Paolo Mannelli and Antoine B. Douaihy were study investigators. Sarah C. Akerman, Abigail Zavod, and Maria A. Sullivan were involved in the study design and data analyses. Anna Legedza conducted the data analyses.

## Data Availability

The data collected in this study are proprietary to Alkermes, Inc. Alkermes, Inc. is committed to public sharing of data in accordance with applicable regulations and laws.
